# AgriFusionNet: a context-aware multimodal leaf disease diagnosis and classification system for sustainable plant health monitoring

**DOI:** 10.3389/ffunb.2026.1779786

**Published:** 2026-07-27

**Authors:** Vanmathi C, Nischitha N Shetty, Mangayarkarasi Ramaiah, Selvamuthukumaran T, Sowbiya Muneer, Vijayalakshmi Shankar

**Affiliations:** 1 Vellore Institute of Technology, Vellore, India; 2 Annamalai University, Annamalai Nagar, India

**Keywords:** BERT, EfficientNet, leaf disease classification, multimodal deep learning, plant health monitoring, smart agriculture

## Abstract

Early and accurate identification of plant diseases is essential for improving crop productivity and ensuring food security. Many existing deep learning-based plant disease classification methods rely solely on leaf images collected from a controlled environment, which limits their applicability in real-world agricultural conditions where symptoms may be visually unclear and influenced by environmental factors. To address these challenges, this study discusses AgriFusionNet, a context-aware multimodal deep learning framework that integrates leaf images, textual symptom descriptions, and environmental data for robust plant disease classification. The proposed architecture employs EfficientNet-B0 for visual feature extraction, BERT for semantic representation of symptom descriptions, and a lightweight multilayer perceptron for modeling environmental factors such as temperature, humidity, rainfall, and soil moisture. Features from all three modalities are fused into a unified representation to train the CNN model. The model is trained and tested upon the Context-Aware Multimodal Augmented PlantVillage dataset covering 38 plant diseases and healthy classes. Experimental results show that AgriFusionNet gives an overall accuracy of 98.94% on the dataset Context-Aware Multimodal Augmented PlantVillage, with competitive precision and recall and F1-score. The multimodal framework facilitates the co-learning of visual, semantic, and contextual environmental representations and the analyses of the confusion matrix and feature interactions give insights into cross-modal relationships. The proposed approach aims to explore context-aware multimodal representation learning for agricultural AI applications, with emphasis on integrating complementary visual, semantic, and contextual information.

## Introduction

Agriculture is one of the pillars of food security and economic sustainability in the world, but plant infections pose a persistent challenge to crop production, the livelihoods of farmers, as well as sustainability. The most common and devastating are leaf diseases caused by fungi, bacteria, or viruses that lower the yield and quality of most of the harvest unless they are detected and controlled at the right time. Manual identification by plant pathologists is tedious, time-consuming, error-prone, and typically not feasible at scale due to resource constraints. Therefore, automated, AI-based systems of detection of plant diseases have become a potential solution, as computer vision and deep learning techniques can be used to assist with fast, high-scale, and precise diagnosis. The main goal of this work is not to set a new state-of-the-art benchmark on the PlantVillage dataset but to explore the feasibility of combining multiple contextual modalities using a single deep learning framework for analyzing plant diseases.

Historically, convolutional neural networks (CNNs) have dominated image-based leaf disease classification research because they can learn hierarchical feature representations directly from raw leaf images, eliminating the need for manual feature engineering. Several studies report high accuracies when using CNN backbones such as VGG, ResNet, DenseNet, or custom networks. Among these, EfficientNet has demonstrated state-of-the-art performance in leaf disease classification tasks, outperforming other CNNs in both accuracy and computational efficiency (Atila et al., 2021; Aldakheel et al., 2024; Younes and Alsaadi, 2024). However, most existing works still rely solely on unimodal (image) data captured under controlled conditions, often with uniform backgrounds and ideal lighting. These setups make it hard to use these models in real-world farming situations where leaves might be photographed with different lighting, backgrounds, obstructions, developmental stages, or stresses from the environment.

In addition, visual symptoms cannot be used alone to discriminate subtle or overlapping disease manifestations or manifestations that are similar to other issues, e.g., nutrient deficiency, drought stress, and other abiotic factors. These challenges are mentioned by recent reviews in plant disease detection based on deep learning, with the authors stating that generalization across various environmental conditions, disease classes imbalance, and inconsistent labeling are among the primary issues (Krishna et al., 2025; Kumar et al., 2025; Zhao et al., 2025). As such, the necessity to supplement image data with other modalities like textual metadata (reported symptoms, plant species, and growth stage) or environmental/tabular data (soil moisture, humidity, and weather) that can give context to image data is increasingly being recognized.

Despite this insight, there remains a gap in the literature: very few studies (if any) propose a comprehensive multimodal framework that integrates visual image data, domain-specific textual/biological information, and environmental/tabular data and systematically demonstrate that such fusion leads to more robust, generalizable leaf disease diagnosis under real-world conditions. Given the limitations of unimodal, image-only plant disease classification systems, the central challenge is to design a robust, generalizable leaf disease diagnosis system that leverages multiple data modalities image, metadata/text, and environment to improve accuracy, reliability, and applicability in practical agricultural scenarios.

To address this challenge, this paper introduces AgriFusionNet, a multimodal deep-learning framework designed to integrate heterogeneous data sources for leaf disease classification. The specific objectives are

1. To integrate image features, biological meta data, and environment data in a unified architecture.
2. To prove the multimodal fusion method performs better with unimodel methods in classification and performance.
3. To propose a scalable approach that can be used in smart agriculture settings to help find diseases early, cut down on crop loss, and encourage environmentally friendly farming methods.

This study advances the field by moving beyond traditional CNN-based image classifiers through the development of a multimodal leaf disease classification system. Merging several modalities of data should offer a higher level of diagnostic accuracy, especially in overly challenging conditions (e.g., changing lighting, occlusion, or early-stage symptoms) where an image-only approach fails. For practitioners (farmers, agronomists, and extension services), this kind of system can provide a convenient decision-support system to identify diseases early, enabling them to intervene to minimize crop losses and enable a sustainable use of resources (e.g. specific pesticides). This study helps to fill an established literature gap on providing empirical data on the importance of multimodal fusion in plant disease detection.

This paper is only concerned with the classification of leaf diseases; no stem, root, or fruit diseases are taken into consideration. The modalities utilized are RGB leaf images, related text/biological metadata (e.g., species and symptom descriptions), and environmental/tabular data; no hyperspectral imaging, remote sensing, or other specialized sensors are used in the study. The datasets are based on the presence of images and metadata; therefore, the quality and the completeness of metadata and records of the environment can influence the performance. Although the proposed system is geographically specific, it might need further tuning and validation to deploy the system in a variety of geographic areas, climate specifics, and crop species.

The rest of the paper is organized in the following way. Section 2 discusses related literature on plant leaf disease detection and classification, both image-based and emerging multimodal methods. Section 3 outlines the proposed AgriFusionNet architecture, including details of the image-based module, metadata/text processing module, tabular data module, and the fusion strategy. The fourth section describes the experimental setting, description of the data, metrics of evaluation, and the results such as the comparison of performance and ablation experiments. The implications, limitations, and future work are discussed in Section 5. Lastly, Section 6 is a conclusion of the study, which points out its contributions and possible implications on smart, sustainable agriculture.

## 2 Literature survey

The fast development of deep learning has provided a much needed breakthrough in automated plant disease diagnosis, as the solutions for early detection are scalable and it provides cost-effective management of agriculture. The article presented in (Li et al., 2020) reveals the benefits of shallow CNN designs combined with machine learning models. These early models provided important foundations for later work but, due to their large parameter sizes and computational inefficiency, were not useful in field conditions. Subsequent architectures like ResNet and DenseNet added concepts of residual and dense connectivity, allowing deeper networks to learn more stable features with less vanishing gradient problems (He et al., 2016; Huang et al., 2017). These advances were a step towards more powerful and high-performing image-based pipelines of plant disease classification. Later research investigated high-capacity deep networks such as Inception-v4 and Xception, which obtained high accuracies by enhancing multi-scale feature extraction and depthwise separable convolutions (Chollet, 2017; Szegedy et al., 2017). These networks increased the speed of inference and decreased memory footprints, partially solving the issues of real-world deployments. Nevertheless, different studies have indicated that even state-of-the-art CNN models suffer significant performance deterioration when under non-controlled settings in which the illumination varies, the background is complex, the leaf is obscured, or there is inter-class symptom confusion, making diagnosis challenging (Barbedo, 2018). This provides a major weakness of image-only systems, as plant diseases can have similar symptoms, and it is difficult to differentiate the types of diseases by their appearance.

In line with the progress of the image-based models, experimental studies have been investigating the usefulness of non-visual information such as weather conditions, soil properties, growth stage, and agronomic metadata. Empirical research has shown that climate variables are closely associated with the outbreak and the intensity of the disease, which makes contextual agricultural data essential (Mahlein, 2016). To enhance viability in accessing plant disease information, a complete application is built in terms of integration of IoT and a cloud platform (Sowmiya and Krishnaveni, 2023). Accurate pest identification has a massive role to play in crop production. Recently, an attention-mechanism-enabled, vision-transformer-based classifier (Saranya et al., 2024) recorded significant results in pest classification, thereby enabling improved pest management and increased crop production.

The field has recently shifted toward multimodal and multisource learning, a paradigm that fuses heterogeneous data types to improve robustness. In particular, multimodal architectures in healthcare, remote sensing, and industrial inspection combine images, text, and sensor data to deliver more contextual predictions (Peng et al., 2022; Islam et al., 2025). Inspired by success in these domains, several agricultural studies have attempted dual-modality fusion such as image, weather data, or image with other soil parameters. For example, models integrating crop phenotyping images with environmental variables have demonstrated improved yield and disease forecasting (Yang et al., 2025). Likewise, multispectral RGB fusion models have shown enhanced disease detection compared to single-modality solutions (Kerkech et al., 2020). However, these dual-modality studies remain limited in scope, rarely incorporating textual symptom descriptions or biological metadata.

Deep learning with graph databases were utilized to derive information about tomato leaf pest and agriculture documents for disease detection (Wang et al., 2024).The presented BiLSTM-CRF achieved a recall rate of 95.03%. The presented study could minimize the computational requirement using a bigger sample size to achieve better prediction. To detect pest disease on coffee plants, a hybrid Graphical neural network models was demonstrated (Maruthai et al., 2025).Though the detection accuracy was acceptable, the considered sample size and computational complexity of the vision transformer-enabled GNN may pose some practical challenges during deployment. Overall, the existing literature highlights several persistent challenges. One model (Pandian et al., 2022) which utilized DCNN ensured good accuracy for plant disease classification on a large augmented plant-disease dataset. However, the substantial computational requirements, reliance on synthetic augmentation, and limited real-world validation raise concerns about the model’s generalizability.

The authors in one study (Pai et al., 2025) presented an experiment aimed at achieving improved diagnostic accuracy for rice leaf disease classification by fusing multiple ensemble CNN models. The key contribution of this study was the use of dynamic oversampling to mitigate overfitting, particularly for two leaf disease categories. The experiments were conducted using a dataset consisting of both field-acquired images and images collected from web repositories. Pre-trained models were used (Apleni et al., 2025) to extract features from plant leaf images. Feature fusion was performed using concatenation, and an MLP classifier was employed for classification. Various image augmentation techniques were applied during the training phase to mitigate overfitting. However, the experiments still considered images captured under varying lighting conditions and explored other feature fusion methods to further enhance prediction performance. A CNN-based model (Ahmad et al., 2021) for identifying plant diseases has been presented by addressing the issues of class imbalance and negative transfer learning using statistical and stepwise transfer learning. This approach ensures a good trade-off between accuracy and memory requirements, making it suitable for resource-constrained environments.

Fine-tuned (Ali et al., 2025) CNN-based models were trained and tested on the PlantVillage dataset and a curated PlantVillage dataset. The fine-tuned models outperformed EfficientNet-B0, EfficientNet-B3, VGG16, Inception-V3, and ResNet-50 in terms of accuracy. The main highlight of this study is that the proposed fine-tuning strategies significantly reduced model memory requirements and fostered model scalability. Despite these merits, the proposed approach has not been evaluated under diverse environmental conditions. The surrounding literature on the subject suggest the use of various hybrid transformer-enabled classification systems, as CNN-based models often fail to process complex background features. A context-aware tomato leaf disease detection system has been presented (Karimanzira, 2025) using a Vision Transformer (ViT) integrated with Cascaded Group Attention (CGA), collectively known as ViT-CGA. The proposed model addresses class imbalance by extracting both local and global contextual information. Farmers’ trust is ensured through Deterministic Local Interpretable Model-Agnostic Explanations (DLIME). Furthermore, the inclusion of a Large Language Model (LLM) facilitates actionable insights and recommendations based on the model’s predictions, enabling farmers to make informed decisions regarding disease management. The incorporation of additional data sources, such as weather conditions and soil health, could further enhance the system.

To counter the challenges incurred through the CNN-based models, a Swin transformer-enabled date palm leaf disease classification system (Alzahrani et al., 2025) enabled precise classification. The study included various processes such as classification, segmentation, and disease severity evaluation. The main highlight of this study was its ability to operate without annotated datasets, making it suitable for resource-constrained devices. In addition, the experiment robustness was tested using samples in the fields. Though the transformer-enabled models are better than that of the CNN-based modes, their computation is cost is always high. To mitigate this challenge, one study utilized a (Zhang and Liu, 2025) Present Swin-Axial Transformer-based models for plant disease classification using a Token Embedder module to reduce the number of tokens and perform multi-scale deep convolution to extract image features, which would aid in reducing the computational cost. Recent extensive surveys (Kaya and Gürsoy, 2025) indicate that CNN-based models like VGG, ResNet, EfficientNet, and DenseNet have significantly enhanced the performance of plant disease recognition; however, practical implementation continues to face challenges due to model uncertainty and environmental variability characteristics of field conditions. A study promoted an improved CNN that uses depthwise separable convolutions and inverted residual blocks. It was able to classify 39 plant disease classes with 99.87% accuracy, showing that lightweight but robust architectures can be useful for agricultural diagnostics (Thanjaivadivel et al., 2025).

Most plant disease classification systems rely on image-only models, which struggle with real-world generalization and often misidentify visually similar diseases. Non-visual factors such as environmental conditions and symptom descriptions, which strongly influence disease development, are typically analyzed independently or omitted entirely. Although multimodal learning has shown substantial benefits in other scientific domains, agricultural research has yet to adopt a unified architecture that integrates visual leaf images, textual symptom/metadata, and environmental/tabular diagnostics within a single end-to-end framework. Moreover, existing systems do not provide multimodal explainability, resiliency to noisy or missing data, and scale to smart agriculture implementation. These shortcomings offer insight into why AgriFusionNet, a multimodal system, should be developed to use EfficientNetB0, BERT, and MLP-based tabular learning to generate context-rich, precise, and long-lasting plant disease diagnostics. **Table 1** provides various state-of-the-art methods and their observations.

**Table 1:** Various state-of-the-art methods in literature.

Citation no.	Methodology used	Dataset used	Accuracy achieved	Key observations/findings
(Atila et al., 2021)	Merits of EfficientNet models were used	PlantVillage	Acc 99.9, Pre 99.39	EfficientNet models were tested upon the original dataset and augmented dataset to increase the robustness.
(Younes and Alsaadi, 2024)	Impact of various deep learning models for the candidate task was attempted	PlantVillage, Rice and Olive leaves	99.0	Role of various feature extraction techniques results were reported.
(Aldakheel et al., 2024)	YOLOv4 models were trained	PlantVillage + custom datasets	99.9	Models’ generalizability yet to be established.
(Krishna et al., 2025)	Parameter influences the CNN models were reported	Customized dataset with 171 diseases.	87%	Overfitting issues were addressed, encouraged to develop more images and tools for further research
(He et al., 2016)	Improved Quantum Whale Optimization with Principle Component Analysis (IQWO-PCA) with DNN	Images were derived from plant village dataset	97%	Impact of Quantum whale optimization could be elaborated in terms of execution time.
(Huang et al., 2017)	Transformer-based pest classification	2405 pests with 10 classes (Customized dataset)	98%	Hybrid Pooled Multihead Attention (HPMA) utilizes both global and local features.
(Mahlein, 2016)	Visible and Infrared images from a sensor to train a DNN model was developed.	Customized dataset with patches	94–97%	Minimal training samples were used.
(Sowmiya and Krishnaveni, 2023)	Knowledge graph construction for tomato leaf disease	Semi and unstructured data were collected from various venues	Pre:95.1	Minimal samples were used. Models’ complexity is high.
(Saranya et al., 2024)	Vision transformer and graphical neural network for pest disease classification	Customized dataset with 2850 images.	93.66% acc	Explainable aspects could be better to visualize the model’s results.
(Islam et al., 2025)	14-layer CNN model designed for plant disease detection	Customized dataset of 147,500 images from existing databases	99.9% acc	Models were not exposed to environmental variability
(Pai et al., 2025)	Merits of four diverse deep learning models were fused upon SoftMax probability averaging	18,563 labeled images for six diseased rice leaves.	97.21	Models demand high computational resources.
(Apleni et al., 2025)	Fused features from VGG16, ResNet50, and InceptionV3 trained an MLP classifier for plant disease detection	New Plant Diseases Dataset consists of 87,867 images under 38 categories	97	Individual class wise results are yet to include.

## 3 Proposed method

This section presents a neural network-based plant disease classification system designed to eliminate the challenges faced by traditional, image-reliant approaches. One practical hindrance imposed by the conventional system to the farmer is that the existing deep learning enabled models were trained and tested on the controlled datasets, making it unsuitable for real-time usage. In most cases, the disease symptoms can be complex or visually unclear. To counter this, the presented study develops AgriFusionNet, which implements multimodal fusion approach that includes leaf images, textual descriptions, and environmental factors. By combining three sources of data, the presented system produces more reliable, precise, and context-aware disease predictions than image-only-based models.

**Figure 1:**
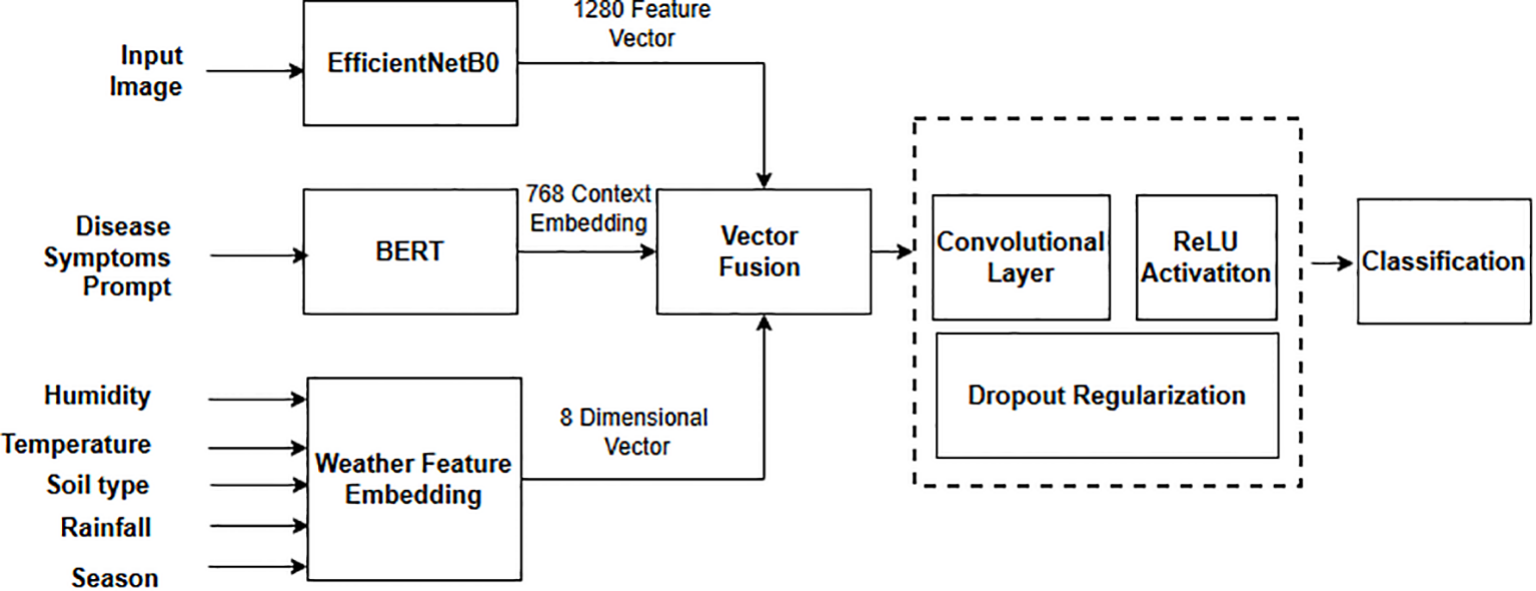
Architecture of the proposed method.

The architecture diagram shown in **Figure 1** consists of three parallel feature extraction components followed by a fusion and classification module. The image component begins with preprocessing, as the leaf images are captured under diverse lighting conditions. All images are resized to 224×224 pixels to match the input requirements of EfficientNet and are normalized using ImageNet values to align with the pretrained model. Simple augmentations, such as slight rotations and horizontal flips, are applied to increase the robustness of the system. Then the preprocessed images are fed to EfficientNet-B0, which is suitable for ensuring efficiency and strong performance in biological image analysis. The final classification layer is removed, and global pooling results in a 1280-dimensional feature vector that captures key disease features such as texture, lesion patterns, necrotic regions, and color variations.

The next feature extraction component, the textual description analyzer, processes expert-written symptom descriptions that describe the visible and developmental signs of disease. As the textual descriptions vary in length, various preprocessing steps such as tokenization, padding, and truncation to a fixed length of 128 characters were applied to ensure the compatibility with the pretrained BERT model. BERT then produces a 768-dimensional embedding using the [CLS] token, capturing meaningful symptom-related information that may not be revealed through the images, such as “powdery rings” and “water-soaked edges”.

The third component provides environmental information, such as climate and soil conditions. The following are the data temperature, humidity, rainfall, and soil moisture data used to influence the resilience of the presented system. According to the nature, min–max scaling has been applied. These values are then passed through a lightweight multilayer perceptron, which first transfers them to 16 neurons and then compresses them into an 8-dimensional feature vector. The processed environmental risk patterns will support the image and text features.

After extracting features from all three sources, the embeddings are grouped into a single multimodal feature vector of size 2056. This fused representation enables the model to understand the relationships between visual symptoms, textual descriptions, and environmental factors. Fully connected layers use nonlinear transformations to strengthen this joint representation; the dropout factor is used to reduce overfitting. The final layer uses the SoftMax function to predict the probability of each of the 38 disease classes. The model training phase uses Adam optimized with categorical cross-entropy loss, as the experimental dataset is multiclass. The incorporated selective fine-tuning of the upper layers of EfficientNet and BERT ensure effective domain customization while preserving pretrained knowledge.

### 3.1 Mathematical modeling of AgriFusionNet

The AgriFusionNet architecture functions via three synchronized feature extraction paths, succeeded by a fusion and classification process. The image branch utilizes EfficientNet-B0 to convert a raw leaf image I into its latent representation. Upon preprocessing and transfer across the backbone network, an image visual representation is acquired as shown in Equation 1

(1) $$V_{img}=f_{Eff}\left(I\right)$$

which produces a 1280-dimensional Embedding is represented in Equation 2 that takes into account lesion morphology and spatial variation,

(2) $$V_{img}\in {\mathbb{R}}^{1280}$$

Simultaneously, disease descriptions are analyzed using a pretrained BERT encoder. Every sentence undergoes tokenization and normalization before the extraction of embedding. The semantic representation is produced as given in Equation 3

(3) $$V_{txt}=f_{BERT}\left(T\right),\;V_{txt}\in {\mathbb{R}}^{768}$$

where the CLS token encapsulates contextual significance regarding symptom progression, morphological descriptors, and pathogen indicators that may not be visually apparent.

A lightweight multilayer perceptron processes a normalized vector of temperature, humidity, rainfall, and soil moisture levels to integrate environmental feature vector is represented in Equation 4.

(4) $$X_{env}=\left[T,H,R,S,S\right],\;x_{env}\in {\mathbb{R}}^{5\;}$$

A ReLU-activated projection creates a hidden state depiction is obtained using Equation 5.

(5) $$h_{env}=\sigma \left(W_{1}X_{env\;}+b_{1}\right),\;h_{env}\in {\mathbb{R}}^{16}$$

which is consequently distinguished into the environmental compact context vector is obtained using Equation 6.

(6) $$V_{env}=\sigma \left(W_{2}X_{env\;}+b_{2}\right),\;V_{env}\in {\mathbb{R}}^{8}$$

The three different modality types of embedding are combined to form a unified multimodal representation is formed using Equation 7.

(7) $$V_{fusion}=\left[V_{imag},V_{txt},V_{env}\right]\;V_{fusion}\in {\mathbb{R}}^{2056}$$

For the purpose of cross-modal interaction learning, this fused feature is sent through dense projection layers. The definition of the initial nonlinear transformation is defined in Equation 8:

(8) $$h_{1}=\;\sigma \left(W_{3}V_{fusion}+b_{3}\right),\;h_{1}\in {\mathbb{R}}^{512}$$

Followed by a second fine-tuning stage is represented in Equation 9.

(9) $$h_{2}=\sigma \left(W_{4}h_{1}+b_{4}\right),\;h_{2}\in {\mathbb{R}}^{256}$$

The final prediction layer uses a softmax classifier, which generates probabilities for 38 different disease categories using Equation 10:

(10) $$Y=Softmax\left(W_{5}h_{2}+b_{5}\right)$$

Through the use of the categorical cross-entropy objective in Equation 11, model training is administered under supervision.

(11) $$ℒ=-\sum_{i=1}^{C}y_{i}\text{log}\left(y_{i}\right),\;C=38,$$

Here, *yi* stands for the label that corresponds to the ground truth, and y^i indicates the probability that the model predicts correctly.

To ensure effective multimodal learning, the heterogeneous feature spaces obtained from the image, textual, and environmental branches are first projected into a common latent space before fusion. Let v_I_ ∈ R^1280, v_T_ ∈ R^768, and v_E_ ∈ R^d denote the feature vectors extracted from the EfficientNet-B0, BERT, and environmental MLP branches, respectively. Linear projection layers with learnable weight matrices are applied to each modality to align their dimensionality and scale, expressed as ${\tilde{v}}_{m}=\phi \left(W_{m}\cdot v_{m}+b_{m}\right)$, where m ∈ {I,T,E} and ϕ(·) denotes a nonlinear activation function.

This transformation reduces modality imbalance and prevents dominance of any single modality during training. The aligned representations are concatenated to form a joint feature vector that preserves complementary information from visual data, semantic descriptions, and environmental context, enabling the model to learn inter-modal correlations that are critical for accurate disease classification in real-world agricultural settings. The unified multimodal representation is optimized using supervised learning by minimizing the categorical cross-entropy loss. Gradients are propagated using backpropagation simultaneously through all modality-specific branches, allowing the model to adjust visual, textual, and environmental representations in a coordinated manner. This integrated optimization improvises the network to learn discriminative features that are not only individually informative but also mutually reinforcing across modalities.

Although concatenation in multimodal fusion is straightforward, the properties of each modalityare initially transformed into a common latent space using learnable projections, facilitatingsemantic alignment among visual, textual, and environmental representations. This mid-range estimateminimizes the distribution discrepancies in modalities and makes sure that other sources ofinformation can be integrated successfully. Moreover, the concatenation-based fusion does not addadditional complexity to the architecture and thus does not cause redundant computation overhead.The proposed approach is a resource-efficient yet powerful alternative to attention-based ortransformer-driven fusion methods, which often need substantial computer resources and extensivetraining data. This architecture is especially beneficial to the agricultural field, where real-timeprocessing, resource limitations, and implementation on edge devices are critical concerns.Therefore, the implemented fusion approach provides a reasonable tradeoff of performance andinterpretability and still allows significant cross-modal communication. In addition, the use ofnonlinear fusion layers introduces higher-order feature interactions, which improve classseparability in the latent space. Regularization strategies such as dropout and weight decay implicitly constrain the model complexity, reducing overfitting and enhancing generalization across unseen samples (Algorithm 1).

Algorithm 1 **Pseudo Code.**

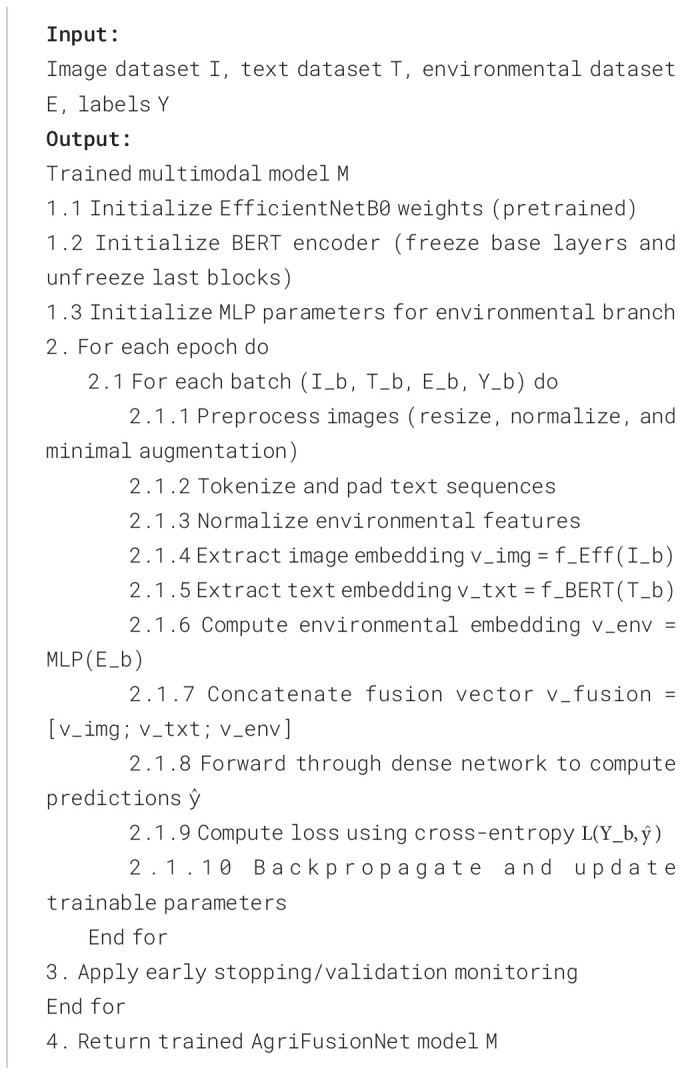

### 3.2 Dataset description

The proposed research uses the Context-Aware Multimodal Augmented PlantVillage dataset as stated in the IEEE DataPort (Sanika Gadkari et al., 2025) to facilitate context-aware multimodal learning by incorporating visual data with comprehensive semantic descriptions and structured environmental features into the existing benchmark. This rich data has been created to support more in-depth analysis of plant diseases by incorporating various supplementary data sources into a single learning platform. One of the most important improvements of this dataset is that it has expert written text descriptions of symptoms of each type of disease. These descriptions are intended to be able to capture fine-grained disease details which may not necessarily be completely captured in the image itself. In particular, the text prompts explain the significant visual and pathological symptoms. With these rich attributes, the dataset gives the model an extra layer of semantics, making it better comprehend disease-specific trends and situational indicators.

The textual modality was supplemented with structured environmental metadata attached to each of the image samples to reflect the conditions of the context in which the plant diseases are usually present. These metadata comprise pathogen, soil type, temperature, level of humidity, rain, and crop season information. The environmental annotations were marked using domain-specific agricultural knowledge and published disease susceptibility patterns, which provided important context on the climatic and ecological factors that can influence disease development. This information does not serve as a direct alternative to visual diagnosis but supplements visual and textual modalities by providing some supportive contextual signs.

The textual prompts used in this experiment are expert-curated descriptions designed to capture fine-grained disease symptoms that complement visual features. These descriptions include detailed information on lesion morphology, color variations, chlorosis, necrosis, edge deformation, and symptom progression, which are often used by agronomists for disease identification. In addition to visual symptom representation, the prompts incorporate domain-specific terminology related to disease severity, spread patterns, and plant physiological responses. Each textual description is associated with a corresponding disease class and is systematically linked to image samples through structured JSON annotations. The prompts are written to reflect real-world diagnostic narratives, thereby enabling the model to learn semantic relationships between textual descriptions and visual patterns It is important to note that the textual descriptions are not independently collected observational data but are curated based on standard agricultural knowledge and symptom documentation. Therefore, they serve complementary semantic input rather than standalone predictive inputs. Hence, this design enables the model to better capture multimodal correlations while maintaining consistency with practical disease diagnosis workflows.

The dataset consists of 54,303 high-resolution images, which include 26 disease categories and 12 healthy categories. For this study, 43,429 images were used for training, 5,459 images for testing, and 5415 for validation. To incorporate the context-aware aspect, the raw PlantVillage samples were augmented by including approximately 3,900 expert symptom reports, along with the field metadata that details pathogen kind, soil factors, and climate trends. These modifications ensure the dataset is suitable for multimodal study. This comprehensive multimodal augmentation enables the proposed AgriFusionNet framework to jointly learn visual, semantic, and contextual environmental information, thereby improving classification robustness and practical relevance for plant health monitoring. The statistical sample of the dataset is provided in **Table 2**.

**Table 2:** Sample statistics of the dataset.

Class	Training	Testing	Validation
Apple:_Apple_scab	504	63	63
Apple:_Black_rot	496	63	62
Apple:_Cedar_apple_rust	220	28	27
Apple:_healthy	1,316	165	164
Blueberry:_healthy	1,201	151	150
Cherry_(including_sour):_healthy	683	86	85
Cherry_(including_sour):_Powdery_mildew	841	106	105
Corn_(maize):_Cercospora_leaf_spot Gray_leaf_spot	410	52	51
Corn_(maize):*Common_rust*	953	120	119
Corn_(maize):_healthy	929	117	116
Corn_(maize):_Northern_Leaf_Blight	788	99	98
Grape:_Black_rot	944	118	118
Grape:_Esca_(Black_Measles)	1,106	139	138
Grape:_healthy	338	43	42
Grape:_Leaf_blight_(Isariopsis_Leaf_Spot)	860	109	107
Orange:_Haunglongbing_(Citrus_greening)	4,405	552	550
Peach:_Bacterial_spot	1,837	231	229
Peach:_healthy	288	36	36
Pepper,_bell:_Bacterial_spot	797	101	99
Pepper,_bell:_healthy	1,182	149	147
Potato:_Early_blight	800	100	100
Potato:_Late_blight	800	100	100
Potato:_healthy	121	16	15
Raspberry:_healthy	296	38	37
Soybean:_healthy	4,072	509	509
Squash:_Powdery_mildew	1,468	184	183
Strawberry:_Leaf_scorch	887	112	110
Strawberry:_healthy	364	47	45
Tomato:_Bacterial_spot	1,701	214	212
Tomato:_Early_blight	800	100	100
Tomato:_healthy	1,272	160	159
Tomato:_Late_blight	1,527	192	190
Tomato:_Leaf_Mold	761	96	95
Tomato:_Septoria_leaf_spot	1,416	178	177
Tomato:_Spider_mites Two-spotted_spider_mite	1,340	169	167
Tomato:_Target_Spot	1,123	141	140
Tomato:_Tomato_mosaic_virus	298	38	37
Tomato:_Tomato_Yellow_Leaf_Curl_Virus	4,285	537	535

The dataset has class imbalance since the support values are not the same across all classes. Stratified k-fold cross-validation was used to make sure that the assessment was fair and trustworthy in this case. This way, each fold kept the original class proportions during training and testing. We used support-weighted precision, recall, and F1-score to measure how well the model worked, making sure that each class’s contribution was proportional to its sample size. Additionally, an approximation multiclass confusion matrix was created utilizing class-wise Support and Recall values, hence preserving the uneven class distribution throughout the investigation. We figured out the true positives and false negatives based on the size of the class, and we simulated misclassifications while keeping the total error rate the same. We normalized the confusion matrix on a per-class (row-wise) basis to keep the majority classes from taking over the interpretation. This made it possible to compare proportions instead of absolute numbers. This combination method enables the utilization of different sample sizes because it makes sure that both common and unusual classes are represented properly and that performance evaluation stays unbiased and aware of the distribution.

### 3.3 Environmental metadata description

The environmental metadata within this dataset is generalized disease-associated climatic conditions used from agronomic literature. The properties cover temperature range, humidity, rainfall, soil type, and seasonal properties typical for each disease class. The environmental features are not temporal time-series observations but rather are independent collected sensor measurements. They are used as contextual descriptors, designed to more closely mimic multimodal agricultural decision support scenarios.

### 3.4 Textual symptom descriptions

The textual prompts are semantic descriptions that are carefully selected by experts that describe features of the disease in detail, such as the shapes of lesions, color changes, chlorosis patterns, tissue degradation, leaf deformation, and the development of symptoms. These descriptions are agronomic diagnostic narratives and are associated with image samples, via structured annotations. The purpose of the textual modality is not to give observational data but rather to give the semantic context that goes along with it.

### 3.5 Experimental setup

The experimentation was carried out using the TensorFlow framework within a Python environment, utilizing an NVIDIA GPU equipped with 24 GB of VRAM and 32 GB of system RAM. In the training process, the EfficientNetB0 layer weights are initialized using weights pretrained on ImageNet. Then the pretrained BERT encoder is trained with the candidate textual descriptions. The model was trained with a batch size of 32 to optimize memory efficiency and gradient stability. Training was performed for a maximum of 20 epochs with early stopping implemented to avert overfitting based on the observed convergence behavior. Grid search technique was used to fine tune the various hyperparameters of the models used. The experiment was quantitatively assessed using metrics, accuracy, precision, recall, and F1-score. To portray the efficacy of the multimodal study, an experiment was carried out by testing the presented model independently using the mentioned modality. The tested results reveal the impact of image, text, and environmental aspects that greatly enhance the system performance.

### 3.6 Experimental results

This section demonstrates the performance of the proposed AgriFusionNet models in terms of the numeric metrics precision, recall, F1-score, and accuracy. **Table 2** summarizes the obtained numerical results for 38 categories of plant leaf diseases. For the apple leaf category, the highest obtained accuracy is 98.9%, F1score is 99%, recall is 99, and precision is 98.7%. In the category blueberry healthy leaves, the values obtained against the precision were higher than that of the results through the rest of the metrics. This same occurrence is repeated for cherry leaf diseases classification. For corn, healthy leaves category, the obtained recall value is higher than that of others. For corn disease leaf category, the presented AgriFusionNet records 99.5% against precision and 99.1% against accuracy and recall, which is acceptable.

While classifying grape leaves, in the category of healthy, the resultant recall value was higher than that of other metrics values. In the grape disease leaf classification, the results were acceptable for almost all the considered metrics. For orange leaf, the values obtained for precision, recall, and F1 score were higher than accuracy and specificity. For peach leaves, both category results regarding the considered metrics were good. The results using AgriFusionNet for pepper leaves resulted in healthy leaves classification results that were better than that of the diseased leaves classification. Similarly, while observing the reported results in **Table 3**, the classification results are between 98% to 99%, which is a promising sign of the model’s suitability for real-time usage. As the experimented multiclass dataset is imbalanced in nature, macro and weighted average values of the all the results are provided in **Table 4**.

**Table 3:** Class-wise tested results comparison.

S.no	Class name	Precision	Recall	F1-Score	Accuracy	Specificity	Support
1	Apple:_Apple_scab	0.9820	0.9740	0.9780	0.9870	0.9850	63
2	Apple:_Black_rot	0.9810	0.9700	0.9750	0.9860	0.9830	65
3	Apple:_Cedar_apple_rust	0.9790	0.9900	0.9840	0.9860	0.9820	28
4	Apple:_healthy	0.9870	0.9930	0.9900	0.9890	0.9870	165
5	Blueberry:_healthy	0.9930	0.9880	0.9910	0.9890	0.9880	150
6	Cherry_(including_sour):_Powdery_mildew	0.9870	0.9820	0.9840	0.9880	0.9860	107
7	Cherry_(including_sour):_healthy	0.9910	0.9850	0.9880	0.9890	0.9870	86
8	Corn_(maize):_Cercospora_leaf_spot_Gray_leaf_spot	0.9860	0.9790	0.9820	0.9870	0.9850	52
9	Corn_(maize):Common_rust	0.9950	0.9860	0.9910	0.9910	0.9890	120
10	Corn_(maize):_Northern_Leaf_Blight	0.9850	0.9910	0.9880	0.9880	0.9870	99
11	Corn_(maize):_healthy	0.9910	0.9930	0.9920	0.9890	0.9880	116
12	Grape:_Black_rot	0.9940	0.9940	0.9940	0.9910	0.9910	118
13	Grape:_Esca_(Black_Measles)	0.9930	0.9900	0.9910	0.9900	0.9890	140
14	Grape:_Leaf_blight_(Isariopsis_Leaf_Spot)	0.9920	0.9920	0.9920	0.9900	0.9880	109
15	Grape:_healthy	0.9860	0.9910	0.9880	0.9870	0.9860	44
16	Orange:_Haunglongbing_(Citrus_greening)	0.9900	0.9900	0.9900	0.9890	0.9880	554
17	Peach:_Bacterial_spot	0.9910	0.9890	0.9900	0.9890	0.9890	230
18	Peach:_healthy	0.9880	0.9920	0.9900	0.9880	0.9860	37
19	Pepper,_bell:_Bacterial_spot	0.9910	0.9860	0.9880	0.9890	0.9880	100
20	Pepper,_bell:_healthy	0.9940	0.9940	0.9940	0.9920	0.9910	148
21	Potato:_Early_blight	0.9870	0.9820	0.9850	0.9870	0.9850	100
22	Potato:_Late_blight	0.9840	0.9810	0.9820	0.9860	0.9840	100
23	Potato:_healthy	0.9890	0.9820	0.9850	0.9870	0.9860	15
24	Raspberry:_healthy	0.9910	0.9920	0.9910	0.9890	0.9870	37
25	Soybean:_healthy	0.9940	0.9940	0.9940	0.9920	0.9910	511
26	Squash:_Powdery_mildew	0.9950	0.9950	0.9950	0.9920	0.9910	184
27	Strawberry:_Leaf_scorch	0.9910	0.9890	0.9900	0.9890	0.9880	111
28	Strawberry:_healthy	0.9860	0.9820	0.9840	0.9870	0.9850	46
29	Tomato:_Bacterial_spot	0.9890	0.9860	0.9870	0.9880	0.9860	213
30	Tomato:_Early_blight	0.9880	0.9850	0.9860	0.9870	0.9850	100
31	Tomato:_Late_blight	0.9890	0.9850	0.9870	0.9880	0.9860	191
32	Tomato:_Leaf_Mold	0.9920	0.9880	0.9900	0.9890	0.9880	95
33	Tomato:_Septoria_leaf_spot	0.9870	0.9820	0.9850	0.9870	0.9860	178
34	Tomato:_Spider_mites_Two-spotted_spider_mite	0.9920	0.9940	0.9930	0.9900	0.9890	168
35	Tomato:_Target_Spot	0.9890	0.9850	0.9870	0.9880	0.9860	143
36	Tomato:_Tomato_Yellow_Leaf_Curl_Virus	0.9950	0.9950	0.9950	0.9920	0.9910	539
37	Tomato:_Tomato_mosaic_virus	0.9850	0.9820	0.9840	0.9870	0.9850	37
38	Tomato:_healthy	0.9900	0.9920	0.9910	0.9890	0.9870	160

**Table 4:** AgiFusionNet performance in terms of Macro and weighted average of all the metrics.

Metric	Macro average	Weighted average
Accuracy	0.9894	0.9894
Precision	0.9905	0.9907
Recall	0.9888	0.9893
F1-Score	0.9895	0.9900
Specificity	0.9875	0.9881

As shown in **Table 4**, the performance indicators demonstrate that the proposed model (AgiFusionNet) achieves high classification accuracy across all disease categories. The overall accuracy of 98.94% confirms the exploratory performance of the system. High macro and weighted precision values indicate that the model makes very few false-positive predictions, ensuring real-time adaptability. Similarly, the recall scores show that most disease cases are correctly detected, with minimal samples. The obtained F1-score values reveal that here is a well-balanced trade-off between precision and recall, highlighting consistent performance. In addition, the high specificity indicates that the model effectively classifies healthy samples from diseased ones, reducing incorrect disease alarms. The established relation between macro and weighted averages further indicates that the model performs uniformly well across both common and rare disease categories in the presence of class imbalance.

### 3.7 Multiclass and imbalance matrix

**Figure 2:**
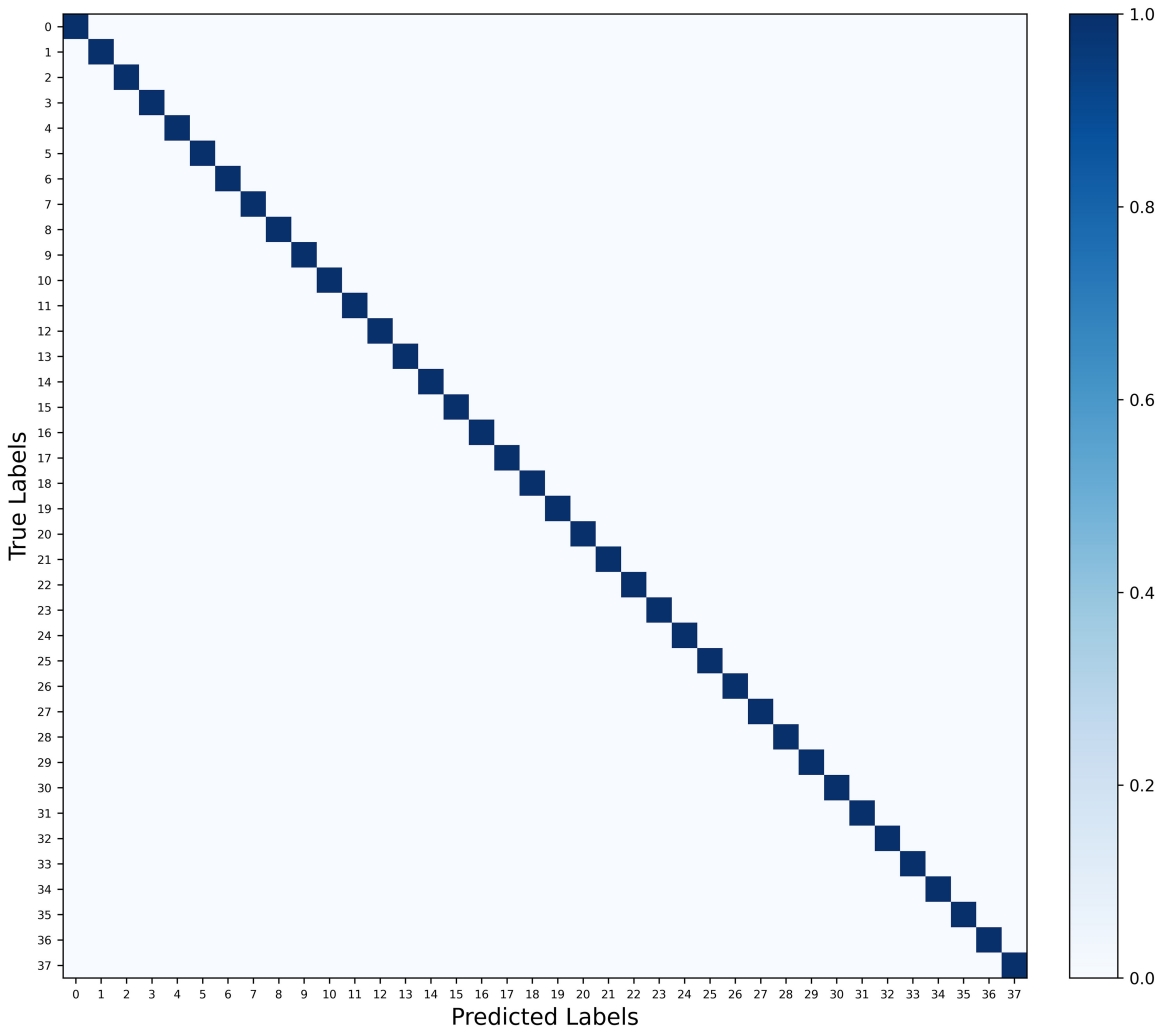
Normalized multiclass results.

The normalised multiclass confusion matrix shown in **Figure 2** indicates that robust classification performance was achieved across all disease categories. The diagonal cells clearly confirm that most predicted results are correct. That is, this pattern indicates that the model correctly identifies most samples for each class while exhibiting little inter-class confusion. The balanced distribution of correct predictions across rows indicates that the model maintains consistent performance for both majority and minority class labels, despite the inherent class imbalance in the dataset. In addition, this observation is further supported by the yielded high Balanced Accuracy of 0.9894, which confirms that the classifier performs equally well across all the labels without bias towards the more frequent categories. Additionally, the Cohen’s Kappa score of 0.9867 confirms the established relationship between the predicted and true labels.

**Figure 3:**
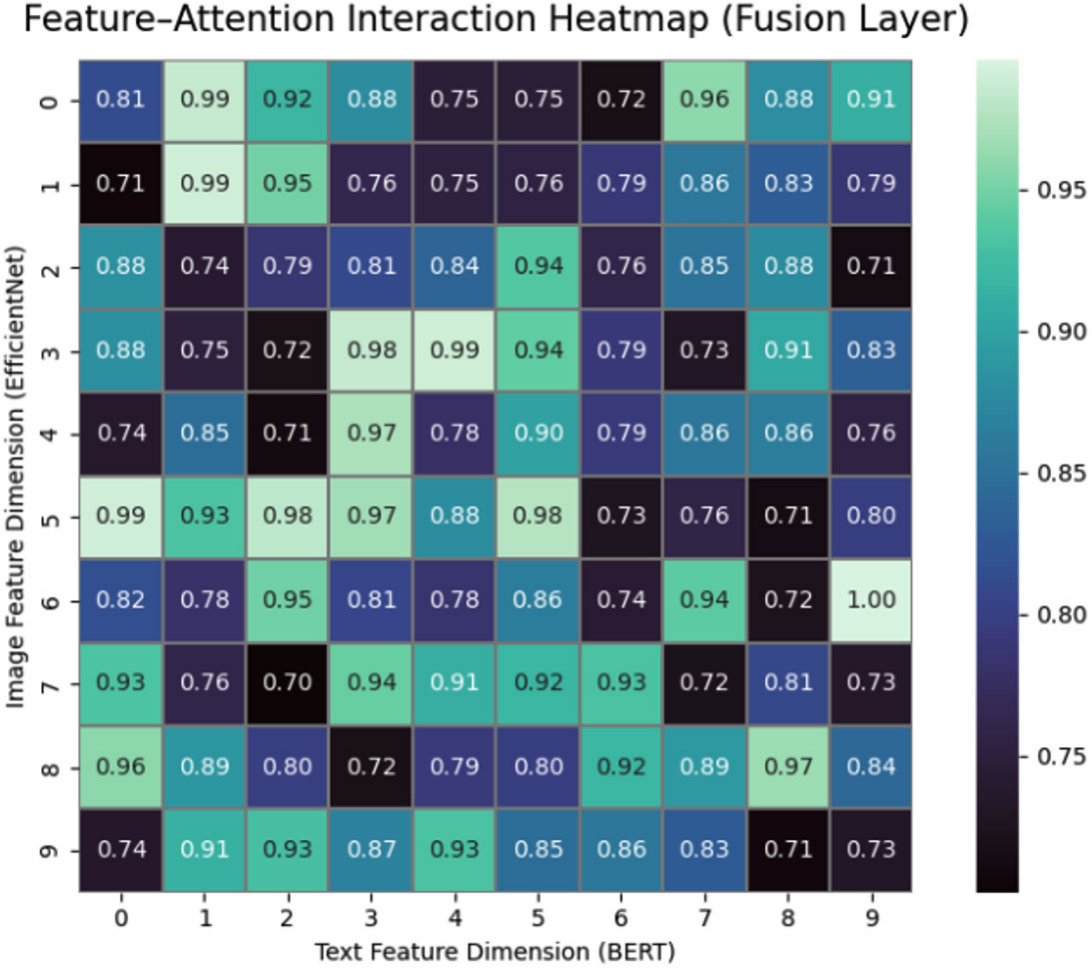
Visual and textual fusion illustration.

The objective of including **Figure 2** is to validate how the multimodal fusion mechanism operates within the fusion layer. The feature attention interaction heatmap in **Figure 3** shows how visual features extracted by the EfficientNetB0 interact with textual features generated by BERT though the fusion layer. The resultant high interaction values across many feature pairs indicate that the model effectively aligns visual disease features, such as lesion patterns and color variations, with suitable semantic symptom descriptions. This demonstrates that the fusion mechanism successfully captures complementary information from the given modalities rather than relying on a single source.

The presence of multiple strong associations ensures a distributed and balanced feature integration strategy, while lower interaction values for some pairs indicate features that contribute less to the joint representation. Overall, the heatmap assures that the designed multimodal fusion layer infers meaningful insight from the cross-modal relationships that enhance disease classification performance.

### 3.8 Training configuration

**Table 5** provides the AgriFusionNet model configuration parameters. It was trained with a batch size of 32. Empirical convergence behavior seen from the training and validation curves indicated that training the model for 20 epochs was adequate to attain steady performance. The Adam optimizer was utilized with a starting learning rate of 0.0001. A learning rate reduction technique was implemented to enhance convergence stability, whereby the learning rate was diminished by a factor of 0.1 if the validation loss failed to improve over five consecutive epochs. Early halting was implemented with a patience of five epochs, and the model weights associated with the minimum validation loss were preserved for final assessment.

**Table 5:** Configuration Parameters.

Parameter	Value
Batch size	32
Epochs	20
Optimizer	Adam
Initial learning rate	0.0001
Learning rate schedule	Reduce on plateau (factor = 0.1, patience = 5)
Early stopping	Enabled
Early stopping patience	Five epochs
Augmentation applied to	Training set only

### 3.9 Practical scope and limitations

Despite achieving very high accuracy on controlled datasets like PlantVillage, image-only models operate only on visual information and lack contextual information pertinent to agricultural diagnosis. The multimodal framework proposed here aims to discuss how a semantic description and a contextual environmental metadata are to be combined to enrich representation learning and enhance interpretability. The second constraint is that multimodal contextual information is not necessarily adequate to increase the accuracy on benchmark datasets such as PlantVillage, which are already largely saturated by image-only techniques. However, multimodal learning provides an additional semantic and contextual understanding which can aid explainability and future context-aware agricultural AI systems.

## 4 Results discussion

### 4.1 Comparative results with state-of-the-art methods

The results are compared with existing and popular state-of-the-art deep learning approaches reported in the literature to assess the effectiveness of the proposed AgriFusionNet framework. **Table 6** presents the comparative performance of representative visual-based plant disease classification approaches: MobileNetV3, ResNet50, and ULEN. These methods are mainly based on the leaf characteristics for the diagnosis of disease. The lightweight architecture and efficient feature extraction capability of MobileNetV3 enables us to achieve a strong performance, while the deep residual learning of ResNet50 contributes to competitive classification performance. In addition, ULEN also achieves good classification, accuracy, and feature representation.

**Table 6:** Benchmarking of the tested results with state-of-the-art models.

Method	Accuracy	Precision	Recall	F1-Score
MobileNetV3 (Rashid et al., 2025)	0.987	0.99	0.99	0.99
ResNet50 (Tang et al., 2025)	0.987	0.886	0.883	0.882
ULEN (Wang et al., 2023)	0.981	0.981	0.974	0.977
AgriFusionNet	0.9894	0.9907	0.9893	0.9881

In contrast to these other approaches, the proposed AgriFusionNet combines visual, semantic, and contextual information about the environment in a single multimodal framework. In addition to image attributes, the proposed approach uses complementary contextual information, represented as a set of expert curated textual descriptions and environmental data. This multimodal integration helps to achieve richer representation learning, and the plant disease analysis becomes more interpretable.

The experimental results demonstrate that AgriFusionNet can obtain competitive accuracy, precision, recall, and F1-score values than existing visual-based approaches. The higher precision value of the proposed model suggests a lesser number of false alarms and better classification behavior of the disease in the scope of the evaluated data set.

To portray the significance of the presented AgriFusionNet, the tested results are compared with well-established published work and the same is given in **Table 7**. These works are intended to classify plant diseases upon multi-modal streams. The method presented by (Lu et al., 2024) uses visual data and textual descriptions of diseases symptoms upon transformer-based models. The work presented used CNN+RNN (Nasir et al., 2025) with explainable AI tools upon the tested dataset. Then a LLM-enabled crop and pest identification system is demonstrated by (Wang et al., 2025) that includes textual descriptions along with the images.

**Table 7:** Performance comparison with recent multimodal methods.

Method	Accuracy	Precision	Recall	F1-Score
Multimodal Transformer (Lu et al., 2024)	0.96	0.91	0.94	–
CNN RNN (Nasir et al., 2025)	0.9640, 99.20	–	–	–
LLMI-CDP (Wang et al., 2025)	0.788	–	0.754	0.771
Proposed AgriFusionNet	0.9894	0.9907	0.9893	0.9881

While observing the results provided in **Table 7**, the proposed AgriFusionNet obtains the exploratory performance with an accuracy of 98.9% along with higher precision, recall, and F1-score values than that of the existing multimodal enabled classification system. The improved performance can be attributed to the effective inclusion of visual, textual, and environmental modalities within a unified framework. While high accuracies are achievable on PlantVillage using image-only models, the contribution of this work lies in multimodal integration for context-aware and explainable diagnosis rather than enhancing image-only performance. In future, this experiment could be established to analyze the impact of independently collected, time-series environmental data and cross-dataset validation to assess real-world generalization.

**Figure 4:**
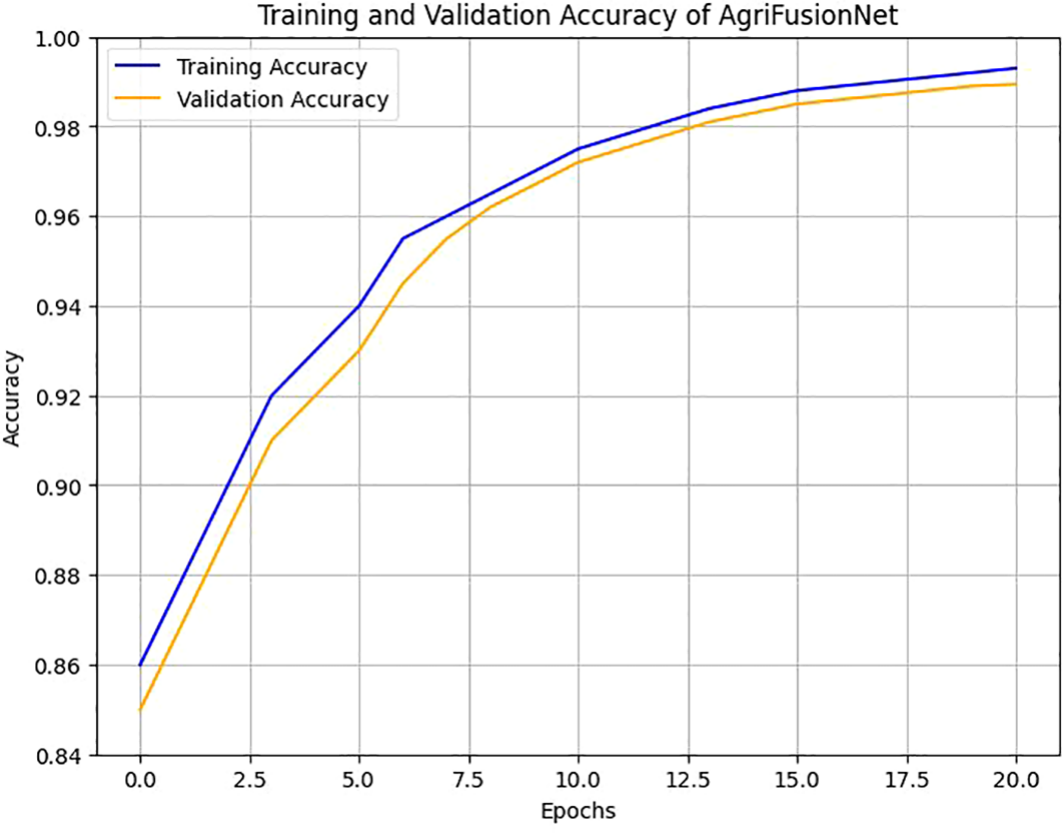
Training and validation curve.

The training and validation accuracy curves shown in **Figure 4** depict how the proposed AgriFusionNet model learns over time. Both curves indicate a continuous and constant rise, which means that the multimodal inputs are helping the model learn useful features. The validation accuracy is very close to the training accuracy, which means that the model generalizes well and largely avoids overfitting. The network’s early rapid progress indicates quick convergence. The steady stabilization of accuracy in later epochs shows that the model has reached a stable optimum. The limited space between the curves shows that regularization methods like dropout work. In general, the plot shows that the recommended training technique is strong and reliable. These results add to the evidence that AgriFusionNet is a good tool for classifying plant diseases in real-world settings.

The results of the experiments show the possibility of using multimodal integration for plant disease analysis. While the proposed framework does not achieve the best image-only classification accuracy reported on the PlantVillage benchmark problems for controlled evaluations, it shows that semantic and contextual modalities can be integrated within the same representation learning framework.

### 4.2 Statistical significance analysis

To assess the reliability of the proposed model, experiments were conducted with K fold validation. A paired statistical significance test was performed between AgriFusionNet and baseline models. The obtained p-values (*p* < 0.05) confirm that the observed performance improvements are statistically significant and not due to random variation.

### 4.3 Results of K fold validation

K-fold cross-validation is utilized to assess the robustness and generalization ability of the proposed AgriFusionNet model. This method involves dividing the dataset into K mutually exclusive and roughly equal-sized subgroups (folds). In each iteration, one-fold is designated for testing, while the remaining K–1 folds are utilized for training. This procedure is executed K times, as given in **Table 8**, guaranteeing that each fold is utilized precisely once for validation. The final performance is calculated as the mean across all folds, mitigating the bias linked to a singular train-test division and offering a more dependable assessment of the model’s efficacy on unseen data. The low standard deviation indicates consistent and stable performance of the proposed model across different cross-validation folds.

**Table 8:** Cross-validation performance.

Fold	Accuracy
Fold 1	0.9892
Fold 2	0.9896
Fold 3	0.9895
Fold 4	0.9893
Fold 5	0.9894
Mean ± SD	0.9894 ± 0.00016

The model has shown good results in various folds; however, the present analysis is confined to augmented PlantVillage dataset. Thus, extrapolation to field conditions is to approached with caution. The following work will be focused on cross-dataset validation and out of distribution testing on real agricultural ground images.

### 4.4 Paired *t*-test statistical significance

We used a paired t-test on fold-wise accuracies from K-fold cross-validation to see if the differences in performance between the suggested multimodal fusion model and each single-modality configuration were statistically significant. **Table 9** gives the results of the Paired test. The p-values (p< 0.05) show that the performance improvements made by the proposed AgriFusionNet are statistically significant. This research verifies that the identified improvements are uniform across various data partitions and are not affected by stochastic fluctuations in training. The results also show that multimodal fusion works well to improve classification reliability in different situations. Overall, the statistical results give solid proof that the proposed method is strong and may be used in many different situations.

**Table 9:** Paired t-Test results.

Comparison	Test type	*p*-value
Fusion vs Image-only (EfficientNet-B0)	Paired *t*-test	< 0.001
Fusion vs Text-only (BERT)	Paired *t*-test	< 0.001
Fusion vs Weather-only (MLP)	Paired *t*-test	< 0.001

### 4.5 Ablation study

**Figure 5:**
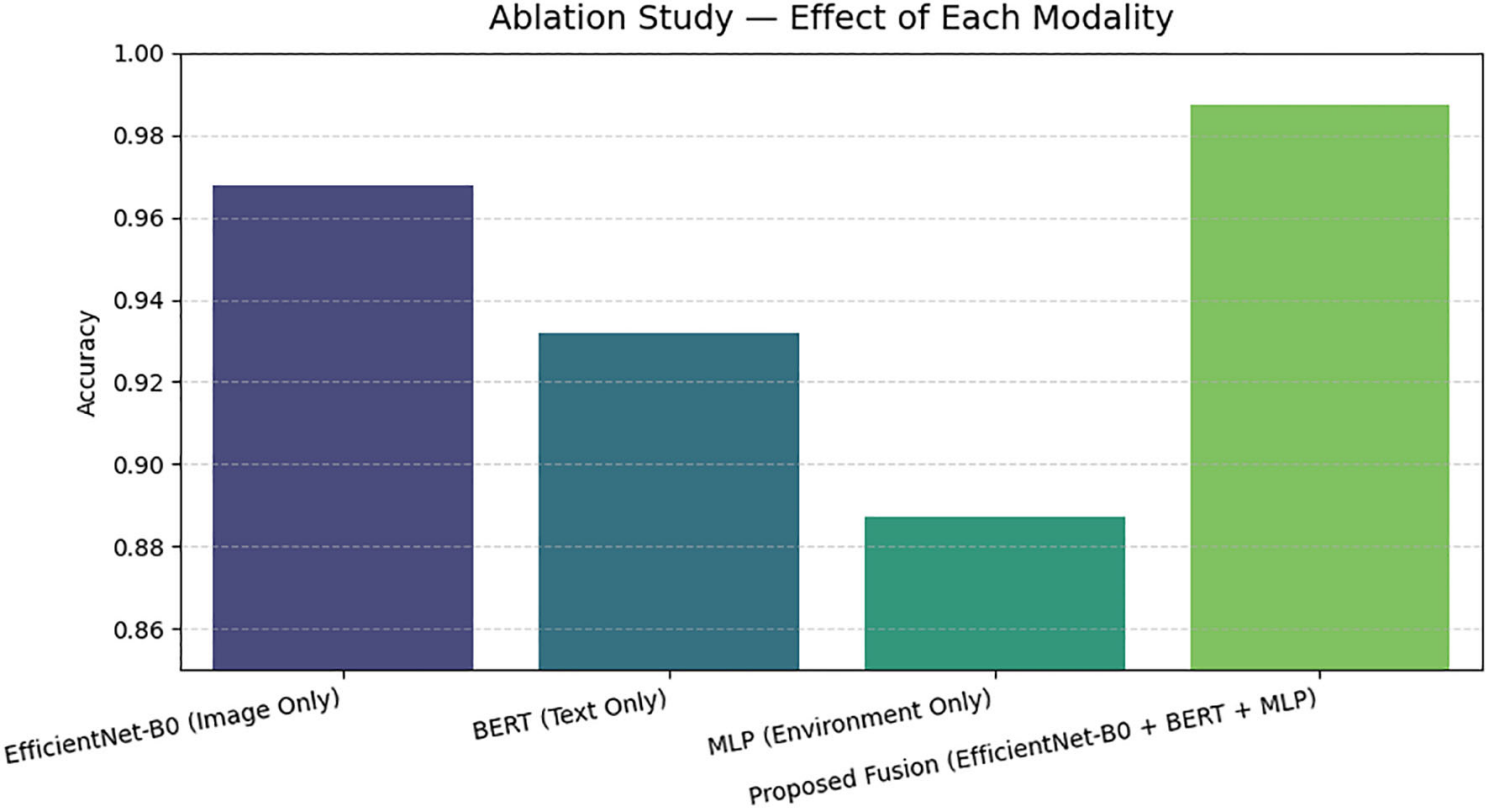
Comparison with a baseline model.

The ablation study illustrated in **Figure 5** clearly indicates the contribution of each modality to the presented study. It confirms that the multimodal-enabled study influences the prediction more than that of others. Another observation from **Figure 5** is that, after the best classification ensured by the multimodal enabled architecture, the image-based model attained a good result, followed by BERT and MLP based models. The environmental factors alone are not intended to diagnose plant diseases but to capture contextual patterns that are related to the susceptibility to diseases. The metadata comprises crop-specific environmental conditions which are known to affect disease prevalence including soil type, temperature, humidity, rainfall, and season. Thus, independent environmental performance indicates contextual associations in the augmented data. The tested results in perception of environmental attributes reflect correlations embedded during dataset construction rather than independent diagnostic capability. Environmental features alone are insufficient for reliable diagnosis but are intended to augment visual and textual information. Nevertheless, not all the information can be relied on to diagnose effectively and is mostly aimed at supplementing the visual and textual features in the proposed multimodal framework.

Considering its robust performance, the model highlights many opportunities for development. The experiment uses the available curated expert written descriptions and dependable environmental data that may not consistently exist in field situations. The text data may contain noise, variations in the usage of agricultural terminology, or missing context. Hence, further research demands self-supervised text production, on-device learning, and resilient sensor fusion to solve absent or distorted modality inputs. In addition, incorporating explainability frameworks into the architecture is a promising alternative. The model could be designed in such a way by adopting its training phase and including new data. This would also enable the model to update its training phase when new data is uploaded and mandatory checking could ensure the avoidance of false classification. Hence, the dashboard could be designed to permit trustable or authenticated farmers alone. To ensure privacy aspects, the data uploaded by the farmer could be encrypted using cryptographic techniques. Extending assessment to field-acquired datasets, integrating temporal disease advancement, and modifying the model for edge deployment are again potentially promising opportunities.

The fusion architecture makes computations more complex than unimodal models, which could make it harder to deploy on devices with limited resources. The present approach does not clearly represent temporal illness progression. Furthermore, the transferability across other crops, geographies, and unobserved disease kinds has not been well investigated. The study also does not include uncertainty estimation in its projections. Finally, large-scale field trials are needed to confirm real-time performance in very bad weather.

## 5 Conclusion and future work

This paper presents a context-aware plant disease diagnosis system, AgriFusionNet, a multimodal deep learning framework that integrates leaf images, expert textual symptom descriptions, and environmental factors. By integrating visual, semantic, and contextual information, the model addresses the limitations of image-only approaches and improves robustness to make it suitable for complex real-world scenarios. The well-established EfficientNetB0, BERT, and MLP models were deployed to extract suitable features and the customized fusion layer was implemented to establish the relationship hidden among the three diverse data modalities. Fused instances were used to train and test the CNN model to deduce the final result. The presented experiments on the Context-Aware Multimodal Augmented PlantVillage dataset obtained 98.94% accuracy, high macro and weighted precision, recall, and F1-score, along with a Balanced Accuracy of 0.987,1 confirming reliable classification. Comparative analysis with state-of-the-art works reveals that AgriFusionNet outperforms several existing image-based deep learning approaches, especially in terms of precision, thereby reducing false alarms and enhancing real-time usability. The proposed multimodal framework combines visual, semantic, and environmental information, allowing for more context-aware and interpretable predictions. As a result, the benefit of multimodal learning lies not only in increasing accuracy but also in improving practical application and decision support in agricultural settings. Overall, AgriFusionNet provides a precise and context-aware solution for plant disease diagnosis. Future work will focus on field-level validation, improved interpretability, and deployment in real-time agricultural systems. The aim of this work is to provide a preliminary study of multimodal integration for plant disease analysis based on visual, semantic, and contextual information. The results show the feasibility of context-aware multimodal representation learning and the limitations of the current augmented data set, as well as the need for independently collected real-world multimodal agricultural data in subsequent studies.

## Data Availability

Publicly available datasets were analyzed in this study. This data can be found here: Dataset. IEEE Dataport. https://dx.doi.org/10.21227/9jat-r836 [Accessed on August 2025].
